# Regulated Fluctuations in Nanog Expression Mediate Cell Fate Decisions in Embryonic Stem Cells

**DOI:** 10.1371/journal.pbio.1000149

**Published:** 2009-07-07

**Authors:** Tibor Kalmar, Chea Lim, Penelope Hayward, Silvia Muñoz-Descalzo, Jennifer Nichols, Jordi Garcia-Ojalvo, Alfonso Martinez Arias

**Affiliations:** 1 Department of Genetics, University of Cambridge, Cambridge, United Kingdom; 2 Wellcome Trust Centre for Stem Cell Research, University of Cambridge, Cambridge, United Kingdom; 3 Departament de Fisica i Enginyeria Nuclear, Universitat Politecnica de Catalunya, Colom 11, Terrassa, Spain; Baylor College of Medicine, United States of America

## Abstract

There is evidence that pluripotency of mouse embryonic stem (ES) cells is associated with the activity of a network of transcription factors with Sox2, Oct4, and Nanog at the core. Using fluorescent reporters for the expression of Nanog, we observed that a population of ES cells is best described by a dynamic distribution of Nanog expression characterized by two peaks defined by high (HN) and low (LN) Nanog expression. Typically, the LN state is 5%–20% of the total population, depending on the culture conditions. Modelling of the activity of Nanog reveals that a simple network of Oct4/Sox2 and Nanog activity can account for the observed distribution and its properties as long as the transcriptional activity is tuned by transcriptional noise. The model also predicts that the LN state is unstable, something that is born out experimentally. While in this state, cells can differentiate. We suggest that transcriptional fluctuations in Nanog expression are an essential element of the pluripotent state and that the function of Sox2, Oct4, and Nanog is to act as a network that promotes and maintains transcriptional noise to interfere with the differentiation signals.

## Abstract 2

The notion that the differentiated state of a cell population is determined simply by expression of specific marker genes is changing. In this work, the authors reveal that a pluripotent cell population comprises cells with temporal fluctuations in the expression of Nanog.

## Author Summary

Embryonic stem (ES) cells are a pluripotent cell population derived from early mammalian embryos. An intrinsic feature of ES cells is their phenotypic heterogeneity: they display promiscuous activation of lineage-specific genes and exhibit a fluctuating flow of differentiating cells. A gene regulatory network (GRN) centred around the transcription factors Sox2, Oct4, and Nanog is essential for the establishment and the maintenance of the pluripotent state. Previous studies had suggested that ES cells can reversibly change their state of Nanog expression without losing pluripotency. Here, we extend these studies by quantifying and monitoring the expression of Nanog in a Nanog-GFP reporter cell line. We show that Nanog levels undergo slow, random fluctuations in ES cells, giving rise to heterogeneous cell populations. We identify two states, one stable, characterized by high levels of expression (HN), and another with low levels of Nanog expression (LN), which is highly unstable. While in the LN state, cells are more likely to differentiate depending on the culture medium. Mathematical modelling shows that a simple excitable system driven by transcriptional noise can account for the observed distributions and behaviours in gene expression. Our study suggests that rather than a discrete state dependent on the fixed expression of a small set of genes, pluripotency is best represented by a state of dynamic heterogeneity of a population driven by transcriptional noise, and that the function of the gene regulatory network centred around Nanog might be to generate dynamic heterogeneities at the population level.

## Introduction

Embryonic stem (ES) cells are cultured pluripotent cell populations derived from the epiblast of mammalian embryos, which can be induced to differentiate into a variety of cell types under controlled conditions [1]–[4]. Most studies with ES cells have been performed on mouse cells, and their pluripotent nature has been demonstrated by their ability to contribute to all tissues of a developing embryo in chimeras [5]. Although derived from the epiblast, the state of the ES cells is similar to that of the inner-cell mass (ICM), and this is reflected in their patterns of differentiation in embryos and in culture [5]–[8]. These observations suggest that ES cells might represent a good experimental system to explore the molecular basis that underlies the establishment and maintenance of different cell fates and their transitions during development.

Traditionally the maintenance of pluripotency in culture requires Leukemia Inhibiting Factor (LIF) [9] and serum or BMP4 [10], as well as the activity of a small, gene regulatory network (GRN) with three core transcription factors: Sox2, Oct4, and Nanog [11]–[13]. Oct4, a homeobox-containing factor, and Sox2, an HMG box protein, bind together at many sites in the genome, including their own promoters and that of Nanog [14]–[16]. Loss-of-function studies show that Oct4 and Sox2 act together to promote self-renewal of ES cells by preventing differentiation [17]–[20]. The levels of Oct4 are particularly critical for the state of a cell: whereas loss of Oct4 results in the loss of pluripotency and differentiation into trophoectoderm (TE), excess Oct4 activity results in differentiation into primitive endoderm (PE)-like cells [21]–[23]. However, these two factors are not sufficient to maintain the pluripotent state, as they cannot act in the absence of LIF. In contrast, when the divergent-homeobox–containing protein Nanog is overexpressed in ES cells, it is sufficient for sustaining pluripotency in the absence of LIF [13],[24], and there is a correlation between its levels and the degree of pluripotency of a cell [25],[26]. Although there are other transcription factors associated with ES cells [27]–[29], a large number of studies support the notion that the trio Sox2, Oct4, and Nanog (SON) is at the heart of a GRN that generates and maintains the pluripotent state. This has been underlined in a number of recent experiments in which these factors play an essential role in the induction of pluripotent stem cells from differentiated cells [30]–[32]. Despite the effectiveness of these transcription factors in promoting and maintaining pluripotency, their mode of action remains unclear.

A number of studies have explored the possibility that there are genes downstream of the SON network that implement the pluripotent state [29],[33]–[36] and, furthermore, that chromatin modifications play a role in the maintenance of this state (reviewed in [37],[38] and [39]–[42]). However, no clear consensus has emerged from these experiments, and there are no clear candidates for establishing and maintaining stemness other than the elements of the SON network and a small, closely associated group of genes [28],[33]. Despite their central role in the definition of the pluripotent state, none of the elements of the SON network are specific to ES cells: Oct4 is expressed in the epiblast, in particular in epiblast cells around the streak, early neural ectoderm, and germ cells [43]–[45]; Nanog is expressed in proximal posterior epiblast (where the streak will form), forebrain, and germ cells [13],[24],[46]; and Sox2 is expressed in the ICM and the epiblast [47], and at the beginning of neurogenesis, Sox2 expression becomes restricted to the neural primordium [48]–[50]. Altogether, these observations indicate that, in the context of pluripotency, the integrated activity of these factors as a network is likely to be more important than their simple presence or absence within a cell.

In contrast to the notion of a well-defined, homogeneous, “pluripotent state” associated with a specific profile of gene expression, ES cells exhibit a high degree of heterogeneity reflected in the variegated expression of some of the pluripotent genes' [51]–[54] promiscuous activation of lineage specific genes [55],[56] and a fluctuating flow of differentiating cells in a cultured population. In addition, when induced to differentiate, only a proportion of cells do so in a stable manner [6],[57]. These observations have led to the suggestion that the foundation of pluripotency might be the active maintenance of a poised state for differentiation, a ground state [58]. Here, we explore this possibility and ask whether the variability characteristic of ES cells correlates with “noise” in the expression of Oct4 and Nanog, i.e., variability in the expression of the genes encoding these transcription factors within a genetically homogenous population [59],[60],[67].

Our results show that a population of ES cells represents a dynamic distribution of related states fluctuating between a stable state of high Nanog expression (HN) and an unstable state of low Nanog expression (LN). We also observe that LN cells are prone to differentiate, and exhibit an increased variability in gene expression as well as low-level expression of differentiation markers. Mathematical modelling shows that a simple network driven by the activities of Oct4 and Nanog, and operating as a noise-driven excitable system, can account for many of the experimental observations and predicts instability of the LN state, which can be experimentally demonstrated. Our results have implications for our understanding of the pluripotent state and for harnessing the potential of ES cells.

## Results

### A Dynamically Stable Distribution of Nanog Expression in ES and EC Cell Populations

A number of studies have shown that Nanog plays a central and dedicated role in the control of pluripotency in cultured ES cells as well as in the embryo [13],[24]. For this reason, we decided to use its expression as readout of the activity of the SON network by using an ES cell line with an insertion of GFP into the Nanog locus (TNGA [25]; see Materials and Methods) and also an embryonal carcinoma (EC) P19 cell line bearing a Nanog promoter fusion to YFP (P19 OTOY cells; see Materials and Methods). In culture, EC cells have properties and expression profiles similar to ES cells ([61],[62] and see Figure S1A) and, despite some phenotypic and developmental differences [61],[62], have provided useful and supporting information about the nature and properties of ES cells [1],[62]. This relationship between the two cell types is a useful tool in the definition of pluripotency because a comparison of their properties will allow us to distinguish essential properties of the system, from some that might be cell line–specific.

**Figure 1:**
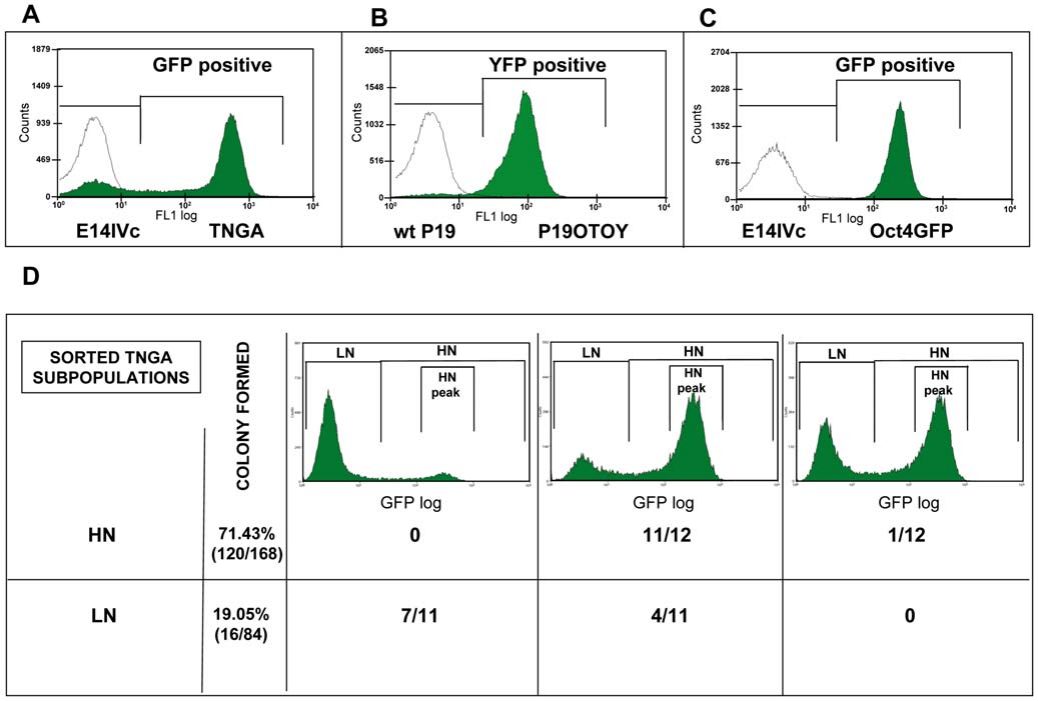
A dynamically stable distribution of Nanog expression in ES and EC cell populations. The steady-state distribution of Nanog reporters (TNGA, P19OTOY) and Oct4 reporter (Oct4GFP) in serum+LIF culture condition (statistical analysis performed using at least four independent experiments, see Material and Methods) (A–C) and distributions generated from single TNGA cells (D). (A)The steady-state GFP expression profile of transgenic TNGA (Nanog-GFP knock-in) ES cell line (green) compared with parental E14IVC cell line (unfilled), which shows the level of autofluorescence in this cell line. TNGA cells exhibit a GFP-negative peak with a mode value of 7.69±2.05, and a GFP-positive peak with a mode value of 391.70±63.49. (B) YFP expression profile of transgenic P19OTOY EC cell line (green) compared with the P19 parental cell line (unfilled). In the serum+LIF condition, 3.14±0.36% of the whole cell population fall in the YFP-negative range (peak with a mode value of 14.37±8.09), whereas 96.90±0.34% are YFP-positive (peak with a mode value of 124.00±25.20). wt, wild type. (C) The steady-state GFP profile of transgenic Oct4GFP ES cell line (green) compared with the parental E14IVC cell line (unfilled). At any given time, only 0.24%±0.08% of the whole cell population is GFP negative, while 99.76±0.08% is GFP-positive (peak with a mode value of 84.97±12.55). (D) GFP-positive (HN) or -negative (LN) single cells were isolated from a steady-state TNGA cell culture and subcultured individually for 11 additional days in normal growth conditions. From the 168 seeded GFP-positive cells, 120 formed colonies; whereas only 16 out of the isolated 84 GFP-negative cells did so. Twelve independent colonies from each experiment were randomly picked and FACS scanned to assess their profiles of Nanog-GFP expression. Regardless of the initial GFP status of the individual cells, when they formed a colony, most of them reconstituted the original dual-peak profile, though the relative proportions of cells in the dual peaks varied. We observed three main types of expression profiles generated by the single-cell–derived clones, which appeared with different frequencies in the case of the two isolated subpopulations, as indicated. The LN cells were somewhat biased towards the original profile, perhaps reflecting that some of them have made a decision to differentiate (see main text).

In steady state, the expression of Nanog-GFP in a population of ES cells is closely related to that of Nanog itself ([25], see also Figure S1B) and exhibits a distribution with a prominent peak in a region of high expression and a smaller and flatter peak, typically about 5%–25% of the total population (depending on the culture conditions), two orders of magnitude lower within the region of autofluorescence (Figure 1A). A related profile, but with a narrower dynamic range and a smaller proportion of cells in the low-expression peak, can be observed for Nanog-YFP expression in P19 OTOY cells (Figure 1B). These distributions have a number of properties:

1. The distributions are gene- and promoter-specific, i.e., a profile is a signature of a particular gene (compare Figure 1A–1C).
2. For a defined set of culture conditions, the profile is reproducible and stable over time, i.e., the shape, median, relative value of the peaks, and dynamic range hardly change when cells are passaged several times (Figure S2).
3. Although culture conditions can and do change the relative distribution of the cells in each of the two peaks, they do not shift substantially the average location of each peak, or the dynamic range of the profile (Figures S2 and S3).
4. The expression of Nanog is not only heterogeneous in cultures of ES cells but, as shown recently, dynamic [25],[51],[53]. Analysis of the interconversion between cells expressing high and low levels of Nanog reveals that cells from either end of the distribution will regenerate the full original distribution after a period of time (Figures S2 and S4).
5. The distribution is a “built-in property” of the cells: single cells from the TNGA cell line, cultured for 19 d, showed a strong tendency to regenerate the original distribution. This is true even for cells from the LN population that begin with Nanog-GFP expression equal to the level of autofluorescence (Figure 1D).

Altogether, these observations indicate that the expression of Nanog in ES and EC cells should not be represented by some average value of expression, but as a distribution of cells with differing levels of Nanog. This distribution exhibits a signature profile characterized by two cell populations, one with high expression of Nanog (HN) and a second one, with fewer cells expressing low levels of Nanog (LN). These two populations are in a dynamic equilibrium, with expression averages and dynamic ranges determined by internal parameters, but with proportions of cells in particular states determined by culture conditions (Figure S3).

### The Position of a Cell in the Distribution Determines Its Developmental Potential

**Figure 2:**
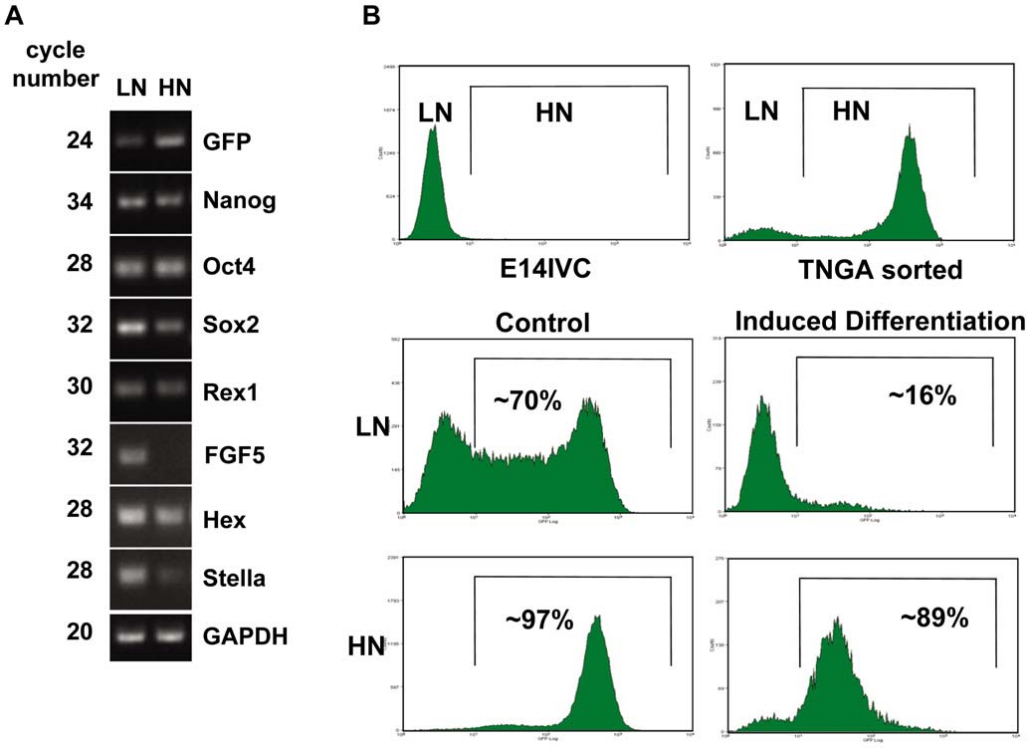
The position of a cell in the distribution determines its developmental potential. TNGA cells with different levels of Nanog-reporter expression (low Nanog, LN, or high Nanog, HN, respectively) have different gene expression profiles ([A] and see main text) and respond differently to the same differentiation cues (B). (A) TNGA ES cells were sorted as LN and HN, based on their Nanog-reporter expression levels, and total RNA was purified. Semiquantitative RT-PCR analyses were performed to detect markers associated with both ES cell pluripotency and differentiation; the transcripts in each population and the cycle number are indicated in the figure. There are no significant differences between the subpopulations in the case of Oct4 expression, but only LN cells express detectable level of *FGF5*, a gene associated with differentiation [89], indicating that the LN population is primed for differentiation. (B) A TNGA ES cell population was sorted into LN and HN subpopulations and subjected to neural differentiation conditions (reduced serum [5%], no added LIF, FGF2 [20 ng/ml] and retionic-acid [RA] [10 µM]) for 3 d. As a control, an aliquot of these subpopulations was kept in culture with 10% serum with LIF. In the case of LN cells, the induced differentiation greatly reduced the number of cells reaching HN state (16% vs. 70%). On the other hand, the differentiation regime reduced the overall number of cells in the HN state (from 97% to 89%) but led to a noticeable shift in the medial fluorescence of Nanog-GFP associated with the differentiation.

The distributions that we observe might represent the equilibrium state of a population without consequences for the fates of the cells. Alternatively, the HN and LN peaks might be steady states with different developmental potentials. The second possibility is supported by the observation that ES cells expressing low levels of Nanog have a high tendency to differentiate [24], and therefore, it might be that the stationary Nanog profile is related to the differentiation potential of the cells. Cells with low levels of Nanog have been reported to express PE markers *GATA4* [53] and *Hex1* (M. Canham and J. Brickman, personal communication), and we find that they also express high levels of *FGF5*, a gene associated with epiblast differentiation (89 and Figure 2A) These results suggest that the LN and HN populations have different developmental potentials and raise the possibility that the LN population represents a pool of differentiation-poised cells [25]. To test this, we sorted LN and HN cells and exposed them to differentiation conditions (Figure 2B and for details, see Material and Methods). Following 3 d of induced differentiation, 84% of sorted LN cells remain in the LN region of the distribution, have lost their capability to form colonies, and show signs of neuronal differentiation (unpublished data). In the case of sorted HN, only 11% of the cells had expression levels characteristic of LN cells. These cells had also lost their capability to form colonies, but we could identify few differentiated cells among them (unpublished data).

Together, these results indicate that an ES or an EC cell population is represented by a dynamically stable distribution of Nanog expression in which the position of a cell is related to its probability of differentiating. A similar situation has been observed for haematopoietic progenitors in that a distribution of expression of Sca-1, a marker for stem cells, correlated with a distribution of probabilities of differentiation [63]. This suggests that these distributions contain information about the state of a cell in a population and might represent a general feature of stem and progenitor cell populations (see conjectures in the Discussion).

### A Model of Pluripotency as a Noise-Driven Excitable System

The previous observations led us to conjecture that in ES cells, pluripotency is associated with a metastable transcriptional network that promotes continuous transitions between quantitatively different states of activity and generates phenotypic variability, both in time and between cells in the population. Networks of this kind have been associated with transcriptional noise in prokaryotes i.e., stochastic transcriptional events at the level of single cells [64],[65], which serves as a way of creating phenotypic variation at the population level as a substrate for selection [66],[67]. In ES cells, variability resulting from transcriptional noise would serve a related function: to keep a subpopulation of ES cells continuously primed for differentiation without a single cell being precommitted to a particular fate for a long period of time. Such priming would be advantageous in situations in which cells must be ready to respond to a diverse range of external signals within a short period of time and commit rapidly to one of several potential fates. A dynamical mechanism that could provide for such transient and stochastically driven priming is excitability.

A nonlinear dynamical system is called excitable if it responds to small perturbations (beyond a certain threshold) with a large pulse, for example, of gene expression, whose shape is mostly independent of the perturbation [68]. In what follows, we introduce a mathematical model based on the concept of excitable dynamics that aims to interpret the experimental results presented earlier and generates new predictions that are tested experimentally. In our case, the triggering perturbation would be provided by gene expression noise, and the excitable pulse would correspond to transient low expression of Nanog.

**Figure 3:**
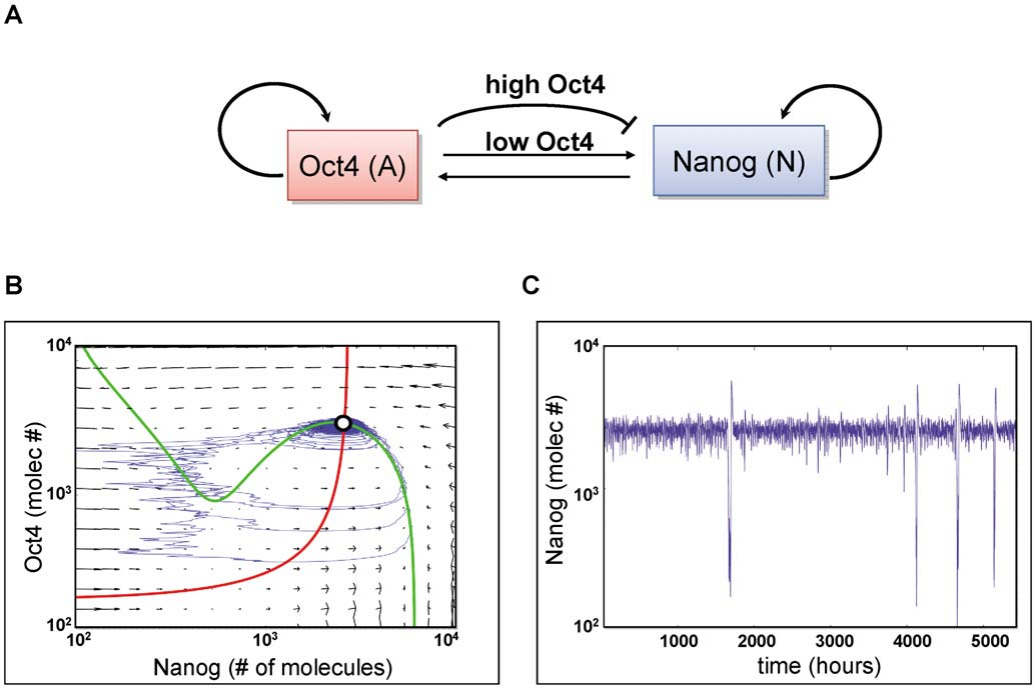
A model of pluripotency as a noise-driven excitable system. A minimal circuit module represents the activity of the pluripotency network as a noise driven excitable system (A–C). (A) This gene circuit contains the known mutual and self-regulatory interactions between Nanog (N) and Oct4 (A), and also includes a negative feedback of the network on Nanog expression. (B) The dynamic behaviour of the GRN underlying this circuit can be traced in phase space. The movement of a cell in phase space is determined by the relative concentration of Nanog and Oct4, and shows that the system has a single stable steady state (white circle), corresponding to high levels of both Nanog and Oct4. The topology of the phase space, as dictated by the location of the N (green) and A (red) nullclines and the slope field (grey vectors in the background), allows that small perturbations of the steady state generate excursions in phase space towards regions where the level of Nanog is low. Since there is no proper steady state in this region, after a deterministic time, the cell is forced to return spontaneously to the state in which Nanog is high, driven by the structure of the network (blue line). A consequence of this dynamical behaviour is that Oct4 levels are more variable in the low-level Nanog state, where trajectories fluctuate strongly along the left branch of the N nullcline, than in the high-level Nanog state, where the cell spends most of the time near the steady state. (C) The corresponding time trace generated after small perturbations of the steady state in the case of Nanog. Following a rare, sudden decrease in the number of Nanog molecules, a very fast return to the HN steady-state level could be observed.

The observations on the expression of Nanog in the population of ES cells (Figures 1D and S4 and [25],[58]) suggest that the LN and HN populations are continuously interconverting and, to a first approximation, could be considered as separate states. Furthermore, the smaller proportion of LN cells (typically 5%–20% of the population) suggests that this state might be a relatively short-lived, transient excitable event, rather than a stable state within a bistable switch. This observation and the known regulatory interactions between Nanog and Oct4 lead us to propose a minimal circuit module (see Figure 3A) to represent the activity of the pluripotency network as a noise-driven excitable system. This gene circuit contains known mutual and self-regulatory interactions between Nanog (N) and Oct4 (A), and we assume that Sox2 is working together with Oct4 in the network [20]. We also include a negative feedback of the network on Nanog expression, representing repression by high levels of Oct4, for which there is some evidence [15],[69] but which could well be assigned to some other heretofore unidentified element of the network. This negative feedback is an essential element of the dynamics of the network.

The dynamics of the GRN underlying this circuit can be represented by a set of two coupled differential equations, given in Box 1, whose behaviour in phase space is governed by the phase portrait shown in Figure 3B. This plot indicates how a cell moves in the phase plane defined by Nanog and Oct4 molecule numbers (see Box 1 and Protocol S1 for a detailed explanation), and shows that the system has a single stable steady state (white circle in the figure), at high levels of both Nanog and Oct4 (which would correspond to the HN state of the distribution). However, the topology of the phase space, as dictated by the location of the N and A nullclines (green and red lines, respectively, in Figure 3B) and the slope field (gray vectors in the figure) allows that small perturbations of the steady state generate excursions in phase space towards regions where the level of Nanog is low (which would correspond to the LN state of the distribution). Since there is no proper steady state in this region, the cell is forced to return spontaneously to the HN state after a determined time, driven by the structure of the network (see blue line in Figure 3B, and the corresponding time traces shown in Figure 3C).

**Box 1. Continuous model of the stemness circuit.**

We consider a genetic circuit (Figure 3A) involving two of the main factors that are known to maintain stemness, namely Nanog (*N*) and Oct4 (*A*). These two factors are known to activate themselves and each other [20]. Furthermore, there is evidence that sufficiently high levels of Oct4 *repress* Nanog expression [14],[33]. A continuous mathematical model that describes the dynamics of this network is:
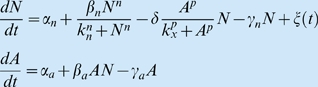
A detailed derivation of this two-dimensional model, including all assumptions made to get to it, is given in Protocol S1. In these equations, *N* and *A* represent the amount of Nanog and Oct4, respectively. Basal expression of the two species are given by *α_n_* and *α_a_*. Nanog self-activation is represented by a Hill function with amplitude *β_n_*, half-maximal activation constant *k_n_*, and Hill coefficient *n*. Oct4 self-activation is assumed to be linear and is modulated by Nanog, with strength *β_a_*. The enzymatic repression of Nanog by Oct4 is described by a Hill function with strength *δ*, half-maximal activation *k_x_*, and Hill coefficient *p*. The two proteins are assumed to degrade linearly at rates *γ_n_* and *γ_a_*. Finally, *ξ(t)* is a Gaussian white noise with zero mean and correlation:
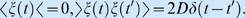

This noise term is used to model heuristically the effect in the circuit of stochastic fluctuations in gene expression. This effect is considered in a more realistic way below, by means of a discrete reaction-based model.

Ignoring in a first stage the presence of noise, the dynamics of this circuit can be determined by plotting the nullclines of the two equations in the phase plane *N*-*A* (green and red lines in Figure 3B). These are lines for which the derivatives of *N* and *A* are zero, and divide regions in phase space where the tendency of growth/decay of *N* and *A* are different. These tendencies are also represented by the slope field (gray vectors in Figure 3B), whose components correspond to the values of the derivatives, in such a way that any deterministic trajectory would be tangent to the vector at that point. The point at which the two nullclines cross (white circle in Figure 3B) is the stable steady state of the system. The parameters giving rise to the behaviour represented in Figure 3B are given in Protocol S1.

In the presence of noise, trajectories eventually escape stochastically from the stable fixed point, forcing the system to perform large excitable excursions towards the region of low Nanog expression (see blue line in Figure 3B and the corresponding time trace in Figure 3C). There is no stable fixed point in that region, which forces the system to return spontaneously back to the HN state after a deterministic time.

In this continuous version of the model, stochastic excursions are generated by a white Gaussian noise term added heuristically to the Nanog equation (see Box 1). Besides establishing a well-defined pulsing dynamics for Nanog, the model also suggests one salient feature of the second component of the network, Oct4. The trajectories shown in the phase-plane portrait of Figure 3B indicate that cells in the HN state spend most of the time near the fixed point of the dynamics (white circle in the figure), and thus the variability of Oct4 is small in HN cells. LN cells, on the other hand, exhibit a much broader distribution of Oct4 levels, and therefore a larger variability in Oct4 can be expected in those cells.

### Dynamics of Nanog Expression in EC and ES Cell Populations

**Figure 4:**
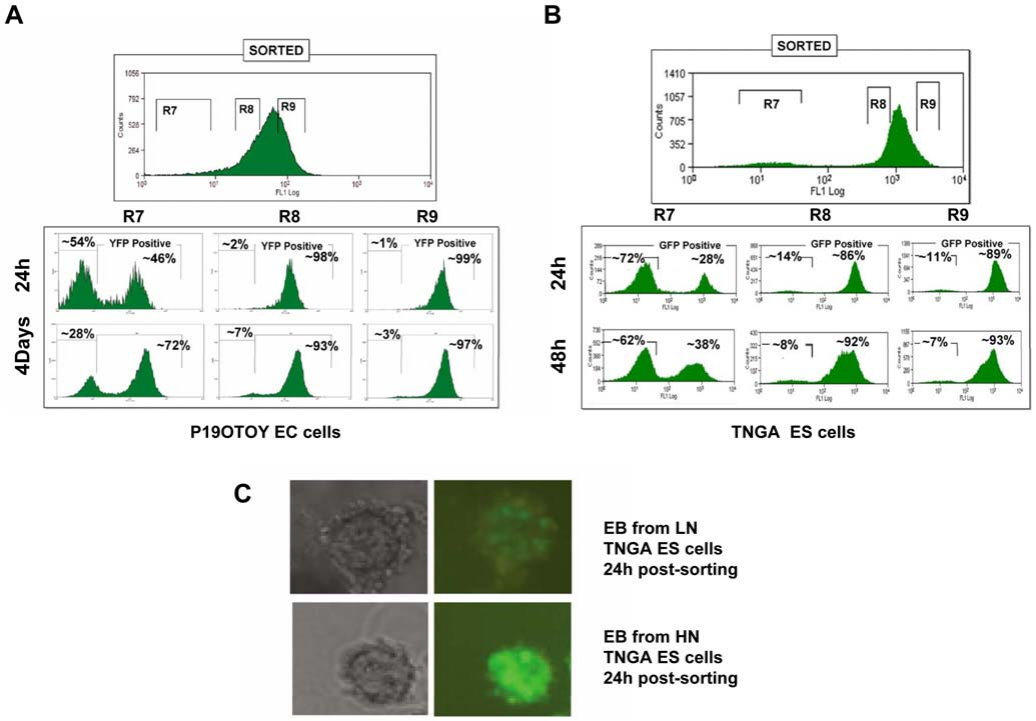
Different behaviour of subpopulations with different levels of Nanog. Dynamics of Nanog-GFP expression in subpopulations of EC (P19OTOY) and ES (TNGA) cells and in embryoid body (EB) expressing different levels of Nanog-FP (A–C). (A) P19OTOY cells with different YFP expression levels (R7, R8, and R9 as indicated) were sorted by FACS. The isolated cells were subcultured for 24 h or for 4 d, and the resulting populations were rescanned. Note that after 24 h, approximately 46% of the formerly LN (R7) cells became positive, whereas after 4 d, approximately 72% of them express higher than autofluorescence level of YFP. In the case of the R8 and R9 (originally YFP-positive, HN) cells, the changes are minor and consistent with the dynamics of the population as a whole. (B) TNGA ES cells expressing GFP at different levels (R7, R8, and R9) were selected and FACS sorted as indicated. The isolated cells were subcultured for 2 d with samples taken every 24 h and rescanned. More than 28% and 38% of the LN (R7) population transit to the HN state in 24 h and 48 h, respectively, whereas less than 8% of R8 or R9 cells became GFP-negative during the same period of time. (C) Embryoid body (EB) formation from isolated LN and HN TNGA ES cells after 24 h. In the case of sorted LN cells, some of them in the EB express GFP, whereas in the case of EB derived from HN cells, the vast majority of the cells maintain GFP expression. The variegated expression in the EB derived from the LN cells is consistent with a stochastic transition from LN to HN.

The model predicts that the LN state is unstable relative to the HN state and that transitions from HN to LN should be rare and stochastic, whereas those from LN to HN should be frequent. This would account for the observed unequal distribution, and imply that at any given time, there will be a pool of LN cells that will exhibit a rapid (deterministic) gain of Nanog expression. We tested this prediction first in a population of P19 OTOY cells by sorting subpopulations of cells expressing different levels of Nanog-YFP and monitoring their ability to interconvert into each other over a period of time. After 24 h, a LN population exhibits a bimodal distribution with about 20%–30% of the cells in a peak centred around the mode value of the HN population of the starting distribution (Figure 4A). In contrast with this unstable behaviour, cells from the HN peak hardly change after 24 h, with about 99% of cells detected in the HN region. We made similar observations in the TNGA cells; in this case, the shift after 24 h is clearer because the dynamic range is larger (Figure 4B), but the essence of the behaviour is the same: a rapid transition from the LN to the HN state. It is noticeable that LN cells do not approach the HN state progressively but rather appear to be choosing between two states. These observations support the prediction from the model that the LN state is unstable and that cells in this state have a high tendency to move to the HN state.

The different behaviour of the HN and LN subpopulations can also be observed in single cells within embryoid bodies (EBs). Sorted LN and HN cells were placed in hanging drops to form EBs and observed for GFP expression after 24 h. Whereas the EBs from the HN population exhibit rather uniform high levels of GFP expression, EBs from the LN population have some cells with GFP expression, corresponding to the cultured population behaviour (Figure 4C).

It could be argued that the stability of the HN state is partly due to the stability of the GFP. Although this might account for the stability after 24 h and make some contribution to our observations, it cannot account for the long-term stability of the state nor to the relative proportions of the HN and LN states in the steady state (Figures 2 and S2).

**Figure 5:**
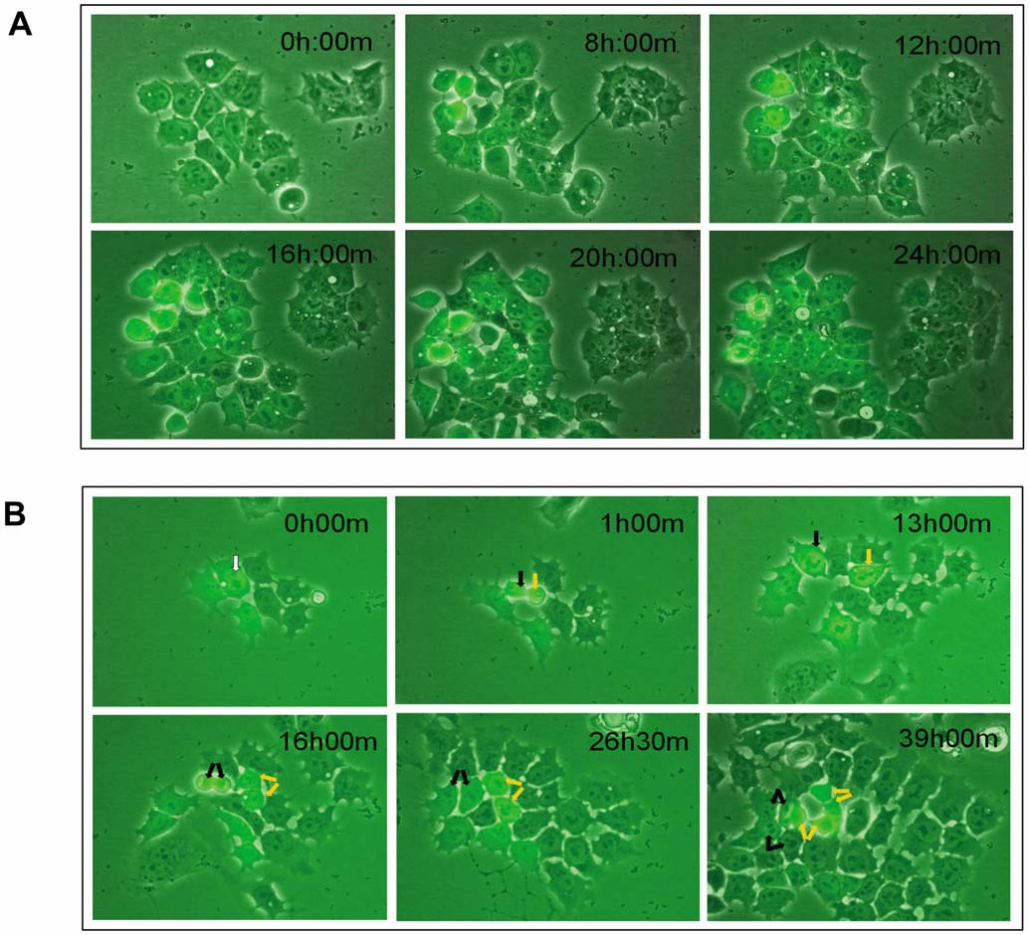
Single-cell dynamics of the transitions between the LN and HN states. TNGA cells from different regions of the distribution were sorted, plated, and then allowed to recover for 24 h before filming. They were then filmed for the indicated periods of time (for details, see Materials and Methods). (A) Sorted LN cells were filmed over 2 d. All cells are initially negative for GFP (at 0 h), but over the course of 24 h, individual cells begin to express Nanog-GFP. Notice that there is no pattern to the onset of expression and that by 24 h, a large proportion of the cells in this cluster are in the HN state. Images are taken from Video S1. Notice that this behaviour accounts for the observations of the experiments referred to in Figure 4B. (B) Similar protocol as in (A) but in this case, cells were selected from the plateau between the LN and the HN states and were filmed over 2 d to reveal the stochastic nature of the decision to move between the HN and LN states. The daughters of the cell labelled with a white arrow at 0 h can be seen to follow different paths: one of them up-regulates Nanog-GFP (yellow arrows), whereas the other down-regulates Nanog-GFP (black arrows). Images are derived from Video S2.

To search for direct evidence of the transitions between the LN and HN states, we filmed sorted LN cells from the TNGA population over 2 d. As expected, LN cells are initially negative for GFP, but over the course of 24 h, it is possible to observe the onset of GFP expression in individual cells (Figure 5 and Video S1). This onset spreads throughout the population. Interestingly, the buildup of fluorescence is not progressive, and cells appear to gain GFP expression rapidly, as would have been predicted from the dynamics of the profiles. An important element of the model that is implicit in the observed behaviour is that the decision to express GFP is stochastic, i.e., there is no spatial nor temporal pattern to the onset of GFP expression. To test this further, we sorted cells from the plateau between the LN and the HN states, which we anticipated might be in an intermediate state of the decision-making process. From a field of largely GFP-negative cells, we observe fluctuations in GFP expression of a stochastic nature (Video S2). Cells from this region of the distribution initially exhibit intermediate levels of GFP, and after 2 d, some of these cells express GFP and some do not, again in an stochastic manner. The nature of the decision is most obvious in the two daughters of the cell marked with a white arrow in Figure 5, which follow different fates: one of them up-regulates Nanog/GFP (yellow arrows), whereas the other down-regulates it (black arrows), consistent with the premises of the model.

Altogether, these observations confirm the instability of the LN state predicted by the model as well as the stochastic nature of the decisions, and suggest that the network introduced in Figure 3 is a good approximation to the real system. They also emphasize the observation that the position of a cell in the distribution is a measure of its fate.

### Heterogeneity of Oct4 Expression

**Figure 6:**
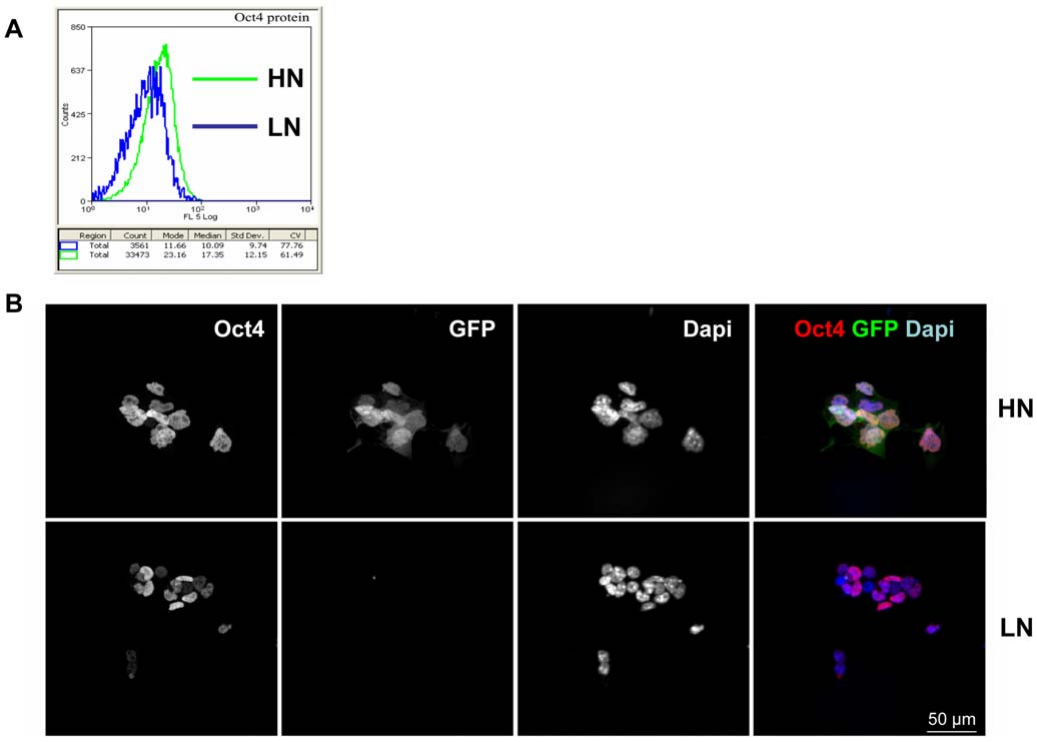
Expression of Oct4 in sorted HN and LN TNGA cells. (A) Expression of Oct4 in TNGA cells sorted for HN (green line) and LN (blue line). Cells were fixed and stained for Oct4 (for details, see Materials and Methods) prior to FACS analysis. Oct4 expression is more uniform and has a higher median in the HN population than in the LN population. (B) TNGA cells were sorted into HN and LN populations and stained for Oct4 (red channel) and DAPI (blue channel). The green channel shows the expression of the Nanog-GFP reporter. The expression of Oct4 is higher and more uniform in the HN than in the LN cells, consistent with the FACS profile shown in (A). There are apparent and reproducible differences in the size of the cells and the nuclei of the two populations. Bar indicates 50 µm.

In addition to the instability of the LN state, the model makes a prediction about the relationship between Nanog and Oct4 expression. It suggests that in the excursions to the LN state, the expression of Oct4 should be more variable than in the HN state (Figure 3B). Observation of Oct4 expression reveals variability, albeit with a smaller dynamic range than that of Nanog, which is also reflected in the expression of GFP under the control of Oct4 (Figure 1C). To test whether there is a correlation between this variability and the state of Nanog expression, we sorted LN and HN cells and stained them for Oct4 expression. Whereas the HN cells have fairly uniform high levels of Oct4 expression, the LN cells show a wide range of Oct4 expression (Figure 6A and 6B). This observation is in agreement with the model and is likely to be correlated with the observation that the LN state is a differentiation-prone state that will require instability of Oct4 expression and function. Furthermore, this observation could be interpreted in terms of our proposal that the LN state arises from excitability rather than as a second stable state, since in the latter situation, one would expect much less variability in the expression of Oct4 in the LN state.

### Stochastic Modelling

In order to compare the population results obtained in the experiments with the dynamics generated by the simple theoretical model described in Box 1, it is necessary to describe in a realistic way the intrinsic fluctuations affecting the network. To that end, we developed a stochastic description of the circuit shown in Figure 3A in terms of a set of biochemical reactions that are compatible with that circuit architecture. Those reactions, together with their corresponding parameters and their relation with the deterministic model of Box 1, are discussed in the Protocol S1.

**Figure 7:**
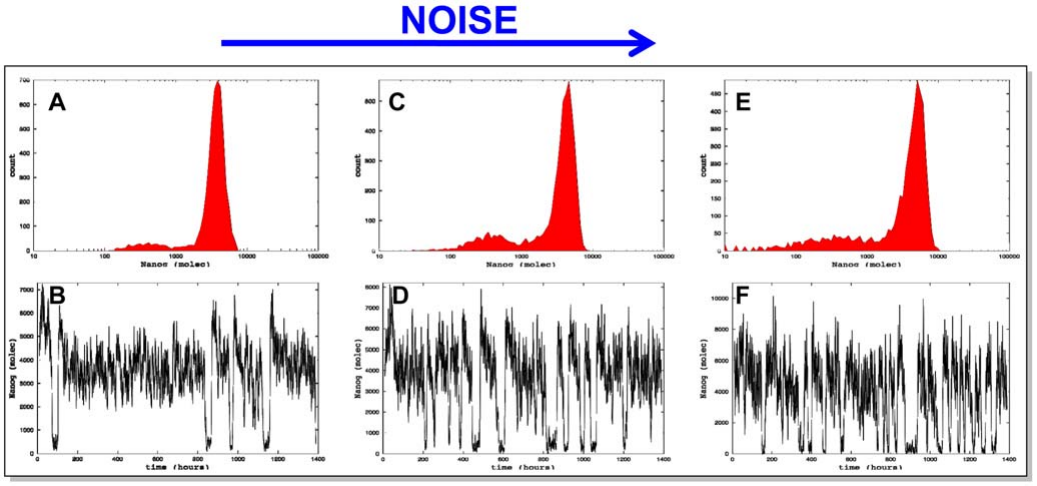
Stochastic simulation of the GRN associated with pluripotency. (A, C, and E) Profiles, resulting from stochastic simulations of the GRN shown in Figure 3, emerging from single cells with increasing levels of noise. (B, D, and F) Trajectories of single cells in the regimes indicated above. For details of the model, see Protocol S1. Notice that increasing the noise (by reducing the effective volume of the cell) increases the width of the peak of low expression.

We simulated the reactions numerically by means of Gillespie's first-reaction method [71] and analyzed the statistics of the resulting dynamical expression profiles of Nanog. Assuming that the system is ergodic, one can equate the dynamical trajectory of a single cell (for a relatively large amount of time) to the expression profile of a population of cells at a given time instant. This allows us to extract a simulated Nanog expression profile from the temporal behaviour of a single realization of the stochastic dynamics of the model. An example of such an expression profile is shown in Figure 7A, with the corresponding time trace from which this profile was extracted displayed in Figure 7B. The profile exhibits features comparable to those observed experimentally, namely two expression peaks, the one at low levels of Nanog expression being much smaller than the one at high levels of Nanog expression. Thus, the dynamics of the population is in agreement with the model of the network and suggests that the instabilities might be due to transcriptional noise.

A simple way to verify this hypothesis is to vary the noise level in a controlled way, and quantify the effects that such parameter change has on the expression profile. We vary the noise globally, by scaling all production rates, together with the rates of the bimolecular reactions [64],[70], in such a way that the noise level is inversely related to the number of molecules of Nanog being produced in the cell (see also Protocol S1). Figure 7 shows the effect of increasing the amount of noise on the Nanog expression profile by reflecting a virtual decrease in the number of molecules of Nanog. The results show that as noise increases, the LN peak increases in size and width (Figure 7A–7C), providing support for the hypothesis that noise regulates the dynamics and occupancy of the two states. They also indicate that the model shown in Figure 3 is a good approximation to the behaviour of the real system and also suggest that noise might not only drive the system, but also tune its performance (Figure 7).

### Sensitivity Analysis of the Network Model

**Figure 8:**
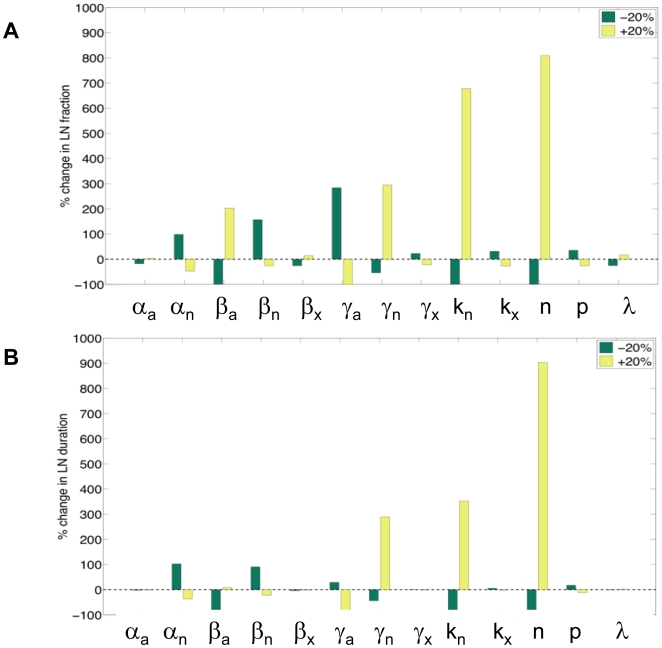
Parameter sensibility analysis of the model. Relative changes in the fraction of LN cells (A) and in the dwell time of cells in the LN (B) with respect to their value in the base excitable regime when all parameters of the deterministic NOS model are increased (yellow) and decreased (green) by 20% over its base (excitable) level. For details of the parameters, see Protocol S1. Changes in the levels of noise are created by altering the effective volume of the cell in the simulation. This results in alterations in the effective concentration of the molecules, which will have an impact on the stochasticity of the biochemical processes. The behaviour of the system is very robust, with a few exceptions highlighted in the figure and discussed in the text.

The experimentally observed sensitivity of the pluripotent state to the levels of Nanog and Oct4 led us to test the robustness of the dynamics emerging from the network by performing an analysis of the numerical sensitivity of the stochastic model to variations in its parameters. To do this, we varied each of the parameters of the model by increasing and decreasing their values 20% off their base level given in Protocol S1. The response of the dynamics of the network to these perturbations was determined by the two features that better characterize its excitable regime, namely, (1) the fraction of low-level Nanog cells with respect to the whole population, and (2) the average duration of the excitable events. Figure 8 shows the relative changes in these quantities (with respect to their base level) when all parameters are changed as described above.

The simulations show that in the model, the excitable regime is maintained over a relatively wide range of parameter values, since LN excursions persist for all but four of the perturbations studied: when β_a_, *k*_n_, or *n* are decreased by 20%, or when γ_a_ is increased by the same amount. In those four cases, both the fraction of cells in the LN state and the average duration of the LN events drop to zero, whereas in all remaining perturbations, these observables have a finite (nonzero) value. In fact, for some of the parameters (such as α_a_, β_x_, γ_x_, *k*_x_, or *p*), the effect of the perturbation is basically negligible. These observations indicate that the most important factors determining the excitability of the system are the positive autoregulation of Nanog (as reflected in the affinity-level *k*_n_ and the cooperativity *n*), the activation of Oct4 by Nanog (through β_a_), and the degradation of both Nanog and Oct4.

These observations highlight the central role of Nanog in determining the dynamics of the network and thereby the pluripotent state. Furthermore they indicate the need for cooperativity in the activity of Nanog, something that is consistent with observations of a requirement for Nanog dimers for the maintenance of pluripotency [71],[72].

## Discussion

A number of studies over the past few years have shown that a network of transcription factors with Sox2, Oct4, and Nanog at the core is the essential cell-autonomous element for the establishment and maintenance of pluripotency in mouse ES cells [16],[20],[34],[73],[74]. Genomic and proteomic studies have sought to shed some light on the function of this network by searching for downstream genes regulated by the components of the network or a role for epigenetic modifications in parallel or these gene regulatory events. However, except for a few genes closely associated with the SON network and which are not specific to ES cells, e.g., *Rex1*, *FoxD3*, and *Sall4*, there is no evidence for an ES-cell–specific stable transcriptome [27]–[29]. Furthermore, despite the existence of special chromatin modifications associated with ES cells, epigenetic factors do not appear to be required for the maintenance of the pluripotent state, nor is there evidence that DNA modifications play a causal role in the maintenance of the pluripotent state ([75] and reviewed in [58]). On the other hand, the search for extrinsic factors involved in pluripotency has identified LIF, FGF, BMP, and Wnt as required elements for pluripotency. However, of these factors, BMP is necessary, but not sufficient, for self-renewal, whereas FGF triggers transitions from self-renewal to lineage commitment [10],[58],[76],[77]. The situation with Wnt signalling is not completely clear yet, but the available evidence suggests that it assists pluripotency as an element of the SON network, although it might not be essential for the self-renewal of ES cells [78],[79].

Recent studies have shed further light on the role of growth factors on self-renewal. ES cells grown in the presence of inhibitors of FGF, MAPK, and GSK3 propagate and self-renew in culture autonomously in a LIF- and BMP-independent manner [80]. This observation suggests that the main function of growth factors might not be to implement self-renewal as a particular state with a specific genetic identity, but rather o create a network of cross-regulatory interactions that prevents differentiation [58],[80]. These observations stress the importance of the cell-autonomous SON-dependent network in the maintenance of pluripotency but also raise questions about its function because even loss of Nanog expression does not abolish pluripotency, although it increases the probability of differentiation [25].

### Pluripotency as a Dynamic Cell State

Our results suggest that pluripotency is associated with a dynamical system revolving around an attractor of a GRN centred on the activity of Sox2, Oct4, and Nanog. This suggestion captures many of the properties of the pluripotent state and is based on our observation that, in the steady state, an ES cell population can be represented by a dynamic distribution of cells with varying levels of Nanog-GFP expression, whose characteristics are resistant to fluctuations and are stable over time, and which can be established by individual cells independently of their origin within the distribution. This last observation indicates the existence of a cell-autonomous mechanism for the generation of the distribution and of attractor states in the system.

Simulations of a simple model of a dynamical excitable system based on known interactions between members of the SON network and driven by transcriptional noise yields the observed distribution of Nanog expression. This suggests that an interplay between deterministic transcriptional interactions and noise are important elements of pluripotency. In our model, the system has a stable attractor in the HN state, but noise-driven fluctuations lead individual cells to stochastic excursions into a transient LN state, from which they are driven back to the HN state in a deterministic manner dictated by the topology of the network. The accumulation of cells in the LN region of the distribution is a consequence of the dwell time of the cells in the low Nanog-GFP expression state, i.e., of the time the network takes to react to the excitable pulse, and does not represent a stable state per se. In an alternative view, the LN state could be construed as metastable, and the system would be characterized by dynamic bistability between a strongly stable (HN) and a weakly (meta)stable (LN) state. However, although we cannot rule out this possibility, in noisy conditions, the LN would not appear as a stable state, and the difference between a bistable and an excitable system would be purely formal. Nonetheless, it is of interest to devise experimental tests of these possibilities because they might have different practical consequences. For the moment, we favour the excitable model whose essence is the dynamic noisy state of the system, which contrasts with other views that have sought to represent the biology of ES cells as a bistable system with self-renewal and differentiation as alternate stable and incompatible states [81],[82].

Silva and Smith have suggested that the pluripotent state represents a ground developmental state, determined by default, which serves as a platform for multilineage decisions [58]. Our results are consistent with this view and provide a formulation for its possible molecular representation in the form of a dynamic equilibrium of the fluctuating levels of Nanog expression. Furthermore, in this equilibrium, the HN and LN states are functionally significant, because in the LN state, but not in the HN state, cells appear to be prone to differentiate (see also [25]). This is underscored by the lower and more variable levels of Oct4 characteristic of this cell population. Thus, rather than being defined by a specific cohort of genes or a landscape of epigenetic marking, stemness/pluripotency might be a state of dynamic cellular heterogeneity whose engine is the activity of the SON network. The function of this network might not be to regulate specific cohorts of pluripotency-specific genes to create a discrete and static state, but rather to use the fluctuating patterns of its elements to generate periodic interferences of continuous differentiation signals that exist in the medium or that might be intrinsic to the ES cells (Figure 7). In this view, the main function of Sox2, Oct 4, and Nanog is to prevent the stabilization of differentiation signals. The signature of an ES cell would thus be a dynamical state driven by transcriptional noise, and exhibiting transient excursions into the LN state in which cells have an opportunity to differentiate, if the conditions allow. If this is indeed the case, it will be difficult to pinpoint a signature transcriptome for a population of ES cells, as such a transcriptome would depend on the culture conditions, which will determine the relative occupancy of the HN and LN states, the dynamics of the distribution, and also the complexity of the LN state. Recent observations on the expression of *Rex1* [83], *Stella* [56], and *Hex1* (M. Canham and J. Brickman, personal communication) in ES cells do indicate that fluctuations in gene expression are indeed a hallmark of these cells. However, at the moment, it is not clear what the correlations are between the different fluctuations nor whether all of them play a role in defining pluripotency.

The situation we have described here for ES cells is reminiscent of that in *Bacillus subtilis* in which, under conditions of environmental stress, cells can either sporulate or enter into a competent state in which they can uptake DNA to mutagenise themselves and so increase their fitness in harsh conditions [64],[84]. The generation of the competent state is mediated by a noise-driven excitable system in the fashion suggested for the SON network. It might be that these networks provide a robust mechanism to generate cells that must be ready to respond to a diverse range of external signals within a short period of time and commit rapidly to one of several potential fates. This is consistent with the classical role of excitability in neural systems, which is to provide neurons with information-processing capabilities [85]. Within a developing embryo, pluripotent cells must be ready to respond, quickly and in a very reliable way, to a variety of signals coming from neighbouring cells according to the spatiotemporal developmental program of the organism, in the same way that neurons in the brain must respond reliably to multiple electrical signals from their neighbours. We conjecture here that, just as in neurons, excitability might provide a very efficient signal-processing mechanism in pluripotent cells. Accordingly, signals propagating through the developing embryo would lead to the necessary chain of differentiation transitions. ES cells in vitro, on the other hand, would lack the developmental signals, but they would still reveal the excitable program through the Nanog excursions described in this paper, that would only lead to differentiation in a random fraction of cells. In that way, the behaviour of ES cells in vitro shows a glimpse of their capabilities within the embryo.

### Perspectives and Conjectures

Recently, Chang et al. [63] have reported a dynamic distribution of states for haematopoietic progenitors with very similar properties to the one we have described here for Nanog. Furthermore in that system, different subpopulations of the distribution also have distinct developmental potential. This, together with our finding that a simple GRN can model the distribution that we observe, suggests that cells have intrinsic state variables that can be measured reliably and which are likely to be related to the activity of specific GRNs. The nature of these state variables will require further analysis, but one could assume, in analogy with statistical physics, that they might be associated with some biochemical “free-energy” potential intrinsic to the underlying networks, and the distributions that we observe might be their reflection (see also [86]). A conclusion from these observations is that transcriptional noise might be an essential element in the generation of heterogeneity in cell populations and that this forms a basis for pattern formation and regulation. This conclusion leads us to propose two conjectures.

Conjecture 1: Cells, particularly in multicellular organisms, have evolved mechanisms dedicated to the generation, maintenance, and filtering of cellular heterogeneity. In the case of ES cells, we surmise that the main and perhaps sole function of the SON network is to generate dynamic heterogeneity and that it does so by operating within a transcriptionally noisy regime driven by excitability: noise generates heterogeneity in a genetically homogeneous population. A corollary of this proposal is that different populations of stem cells might have networks made up of different component elements but with a similar function, the maintenance of stemness by periodically short-circuiting differentiation signals, and thus keeping the system poised for differentiation.

Conjecture 2: The position of a cell in a distribution of stem or progenitor cells is a measure of its developmental potential. This is clear in the case of Sca-1 in progenitor cells [63],[87] and Nanog in ES cells, and suggests that, at least for these types of cells, the trajectories within phase space that determine their distributions are used to limit the number of cells available for differentiation at any given time, ensuring that in the long term, all cells have the same probability of differentiating, thus providing developmental flexibility.

A practical conclusion from these studies is that distributions of key markers as shown here and in Chang et al. [63] can and should be used as quantitative phenotypes that can provide insights into the activity of GRNs and the state of cells.

## Materials and Methods

### ES and EC Cell Culture

E14IVc and TNGA ES cells were a kind gift from Austin Smith's lab and have been described previously [25]. TNGA cells contain a GFP reporter that is fused to the puromycin resistance gene, which is inserted into the Nanog locus. Oct4GFP cells were a kind gift from A. Surani and have been previously described [89]. All ES cell lines were maintained in GMEM (Sigma, G5154) supplemented with 10% FBS (PAA), 1xMEM nonessential amino acids (Invitrogen), 2 mM l-glutamine, 1 mM sodium pyruvate (both from Invitrogen), 100 µM 2-mercaptoethanol (BDH), and 100 units/ml LIF (made in-house or substituted with ESGRO from Millipore) on gelatinised tissue culture flasks. For the TNGA cells, additional treatment with puromycin (1 µg/ml) for three consecutive passages led to removal of any differentiated cells.

Both P19 (ECACC# 95102107) and P19OTOY EC cells were cultured in MEM-α medium (Invitrogen), supplemented with 10% serum (FCS∶FBS 3∶1; Invitrogen) and 1xMEM nonessential amino acids (Invitrogen). The P19OTOY line was derived from the P19 cells, creating a double transgenic cell line. The Nanog-reporter construct was made by cloning a YFP-Hygromycin Fusion protein downstream of the mouse Nanog promoter (−220/+6) (pGL3-Nanog vector kind gift of Wu da Yong [88]).

### FACS Analysis and Sorting

Cells were prepared for FACS analysis and sorting using a Dako MoFlo high-speed cell sorter or Dako CyAn ADP analyzer by harvesting cells using 0.025% trypsin/EDTA (Invitrogen) and the trypsin neutralized in growth medium. Cells were then resuspended in PBS containing 1% FCS, filtered using 300-µm mesh. Live single cells were selected for further analysis on the basis of FSC/SSC/pulse-width characteristics after prior conformation with propidium iodide that these characteristics yielded live single cells.

ES cells that had been trypsinised to a single-cell suspension were kept in medium containing serum and LIF, filtered before sorting on the Dako Cytomation MoFlo High Performance Cell Sorter. A live/dead dye, Topro3 (Invitrogen) was used for both analysis and sorting to select live cells.

The cell sorter was calibrated each time by using fluorescence beads. Parental E14IVc ES or P19 EC cells were used as the negative control because they display a level of autofluorescence; this was used to calibrate the laser intensities so that they were within the range of 10^0^ to 10^1^. Cells with fluorescence levels (GFP, YFP, or RFP) within this range were considered to be negative (e.g., low Nanog/LN), and the converse is true for levels beyond this (e.g., high Nanog/HN).

The sorted cells were reanalysed to check purity of sorting (which was above 98%).

### Induction of Differentiation

TNGA ES cell population was sorted as LN and HN subpopulations and subjected to differentiation conditions with a reduced amount of serum (5%), no added LIF, added FGF2 (20 ng/ml), and retinoic-acid (RA) (10 µM)) for 3 d. Medium was changed every day during the length of the experiment.

### Single Cell Cloning

Single cells (GFP^−^ or GFP^+^) that had been sorted using the Dako Cytomation MoFlo High Performance Cell Sorter into a 96-well plate were left to settle for 2 h before they were examined for the presence of single cells and GFP in order to check the accuracy of sorting. Single cells were then left to grow for 7 d, during which time the clones reached a reasonable size, but necrosis was not visible in the centre of the colony. Clones were expanded further in a gelatinised six-well plate for 5 d before FACS analysis. Capacity to reform undifferentiated colonies was estimated by counting the number of colonies that were recovered from each single cell.

### Embryoid Body Differentiation

TNGA cells were harvested by PBS-based cell dissociation buffer and stained for E-cadherin (Eccd2) antibody and 7AAD before sorting. Sorted E-cadherin^+^, GFP^+^, and E-cadherin^+^, GFP^−^ cells were reanalysed on Dako CyAn ADP analyzer to check purity of sorting. EBs were made from these sorted cells by the hanging drop method [91]. Approximately 3,000 cells were placed in each hanging drop containing serum without LIF and left overnight to encourage differentiation. After 24 h, EBs were harvested.

### Gene Expression Analysis

TGNA and P19OTOY cells were sorted using the Dako MoFlo high-speed cell sorter, 1×10^6^ LN or HN cells (corresponding to R7 and R9 gates in Figure 4) were selected and recovered in PBS. RNA was extracted using TRIzol reagent. Duplicate reverse transcription reactions using SuperScriptIII, either random hexamer or anchored oligo dT primers, were set up; the products from these reactions were then mixed. Analysis of transcript expression levels was undertaken by semiquantitative real-time PCR (qRT-PCR). PCR reactions were set up and divided into 10 equal aliquots; after the first 21 PCR cycles, an aliquot was removed every two cycles. Shown are PCR products from a reaction four cycles after the product was first visible. Details of primers used are available on request.

### Immunohistochemistry

Immunostaining was performed on fixed cells (4% PFA in BBS with 1 mM CaCl_2_, 15 min) washed and blocked for 30 min in BBT-BSA buffer (BBS with 0.5% BSA, 0.1% Triton, and 1 mM CaCl_2_). Cells with primary antibodies were incubated overnight at 4°C at the following dilutions: Nanog (1∶200; AbCam) and Oct4 (1∶100; Santa Cruz Biotechnology). Cells were washed and blocked in BBT-BSA and then incubated with Alexa-conjugated secondary antibodies (1∶200, from Molecular Probes). Vectashield-DAPI was used as mounting medium, and the images were acquired using a Zeiss LSM 510-Meta confocal unit. The images were acquired in the same conditions of laser intensity, gain, and pinhole, and were processed exactly the same way. For the FACS, cells were immunostained using the same protocol, but using PBS as buffer.

### Fluorescence Live Imaging

Previously FACS-sorted TNGA cells were transferred to a gelatinised culture dish in culture medium.

After a day of incubation, the dish was placed inside of a temperature-, humidity-, and CO_2_-controlled Nikon BioStation IM. We have filmed the cells for 50 h, taking pictures every 30 min. The raw data were transformed and presented as AVI videos.

## Supporting Information

Figure S1 **Pluripotency marker expression in P19 EC and TNGA ES cells.** (A) RT-PCR data showing that cultured undifferentiated P19 cells express a set of pluripotency genes (*Nanog*, *Oct4*, *Rex1*, *Sox2*) that are also characteristic of the undifferentiated ES cells. RNA was extracted from P19OTOY cells and gene expression analysed using a two-step semiquantitative RT-PCR reaction (for details, see Materials and Methods). The expression levels of pluripotency markers are shown. Expression of GAPDH (cycle 28), GFP (cycle 32), Nanog (cycle 36), Oct4 (cycle 32), Rex1 (cycle 40), and Sox2 (cycle 32) were detected. (B) Correlation of Nanog expression and Nanog-GFP expression in TNGA ES cells. HN and LN cells were sorted and stained for Nanog expression (red). Although there is some expression of Nanog in the LN population, it is more heterogeneous and lower than in the HN population. The green channel shows the Nanog-GFP reporter, and the blue channel shows DAPI staining.
(0.75 MB TIF)

Figure S2 **Recovery and stability of the TNGA ES cell population following puromycin selection.** (A) Profile of two consecutive passages from a steady-state population of TNGA ES cells grown for several weeks without puromycin treatment. Notice that under these conditions, cells accumulate in the LN. Compare with Figure 1A. (B) Cells from the culture shown in (A) were treated with puromycin (left column) for three successive passages. The treatment selects for cells expressing high levels of Nanog-GFP and results in the elimination of cells that do not express Nanog-GFP. After three passages, puromycin selection was removed, and Nanog-GFP expression was monitored in the population over eight consecutive passages by flow cytometry. Notice that following the puromycin treatment, the majority of the cells in the culture are in the HN peak (99.96%) and that the relative ratio of LN cells progressively increases until the third/fourth passage, when the distribution stabilizes with a LN population between 15%–25% of the total. This distribution is comparable to the one noted in Chambers et al. [25] (and see Figure 1) and persists for four more passages (P5–P8) thereafter, suggesting that this is a steady-state distribution. For comparison, we show the culture from (A) grown in parallel, without selection in the same medium the cells were before the selection (right column). Long-term culture without selection leads to the increase of cells in the LN peak.
(0.95 MB TIF)

Figure S3 **Culture conditions determine the ratio of LN/HN subpopulations in TNGA ES cells.** FACS profiles (GFP expression) of TNGA cells grown in serum-free (LIF+BMP in N2B27) (left column) or in serum+LIF (right column) culture condition during four passages. In the serum-free condition, only 3%–4% of the whole population of TNGA cells is in the LN state, whereas in serum-containing medium, the proportion of cells with a low level of Nanog expression varies between 22% and 33%. Notice the stability of both profiles over time (steady state) and the similar position of the LN/HN peaks between the two conditions.
(0.43 MB TIF)

Figure S4 **Reconstitution of the distribution of Nanog/YFP expression from the outliers in P19OTOY cells.** LN (R7) (autofluorescence level of YFP expression) and HN (R9) (high level of YFP expression) subpopulations of P19OTOY cells were FACS sorted and subcultured in serum with LIF-containing medium. Periodically, FACS profiles of the samples were taken. Two days after culture, the LN population exhibited a clear bimodal distribution. Over time, it is possible to see how the population evolves towards the original distribution in which the LN peak is diffused by the tail of the HN peak, perhaps reflecting the existence of an occupied transition state between the HN and the LN peaks. By day 8 and certainly by day 10, one can see the population has equilibrated. We do observe some variability in the definition of the LN peak in the P19 cells, highlighting that the dynamic range is an important variable in the definition of the states.
(0.34 MB TIF)

Protocol S1 **Details of the continuous (deterministic) and stochastic (discrete) models.**
(0.07 MB DOC)

Video S1 **Stochastic transitions from the LN to the HN states.** All previously sorted LN TNGA cells are initially negative for GFP, but over the course of filming, individual cells with increased level of GFP expression have been observed. The video shows the first 24 h of this transition.
(3.44 MB AVI)

Video S2 **Stochastic transitions between the HN and LN states.** Sorted cells from the plateau between the LN and the HN states were filmed for 50 h. The stochastic nature of the decision is most obvious in the two daughters of the cell marked with a white arrow at 0 h in Figure 5B. One of them up-regulates Nanog/GFP (see yellow arrows in Figure 5B), whereas the other down-regulates it (black arrows in Figure 5B). The video shows the first 39 h of this process.
(4.80 MB AVI)

## Abbreviations

EB: embryoid body
EC: embryonal carcinoma
ES: embryonic stem
GRN: gene regulatory network
HN: high Nanog expression
LN: low Nanog expression
SON: Sox2, Oct4, and Nanog
