# Supplementary figures and images for: Regulated Fluctuations in Nanog Expression Mediate Cell Fate Decisions in Embryonic Stem Cells
Source: PLoS Biol. 2009 Jul 7;7(7):e1000149. doi: 10.1371/journal.pbio.1000149 (PMC2700273; doi:10.1371/journal.pbio.1000149)

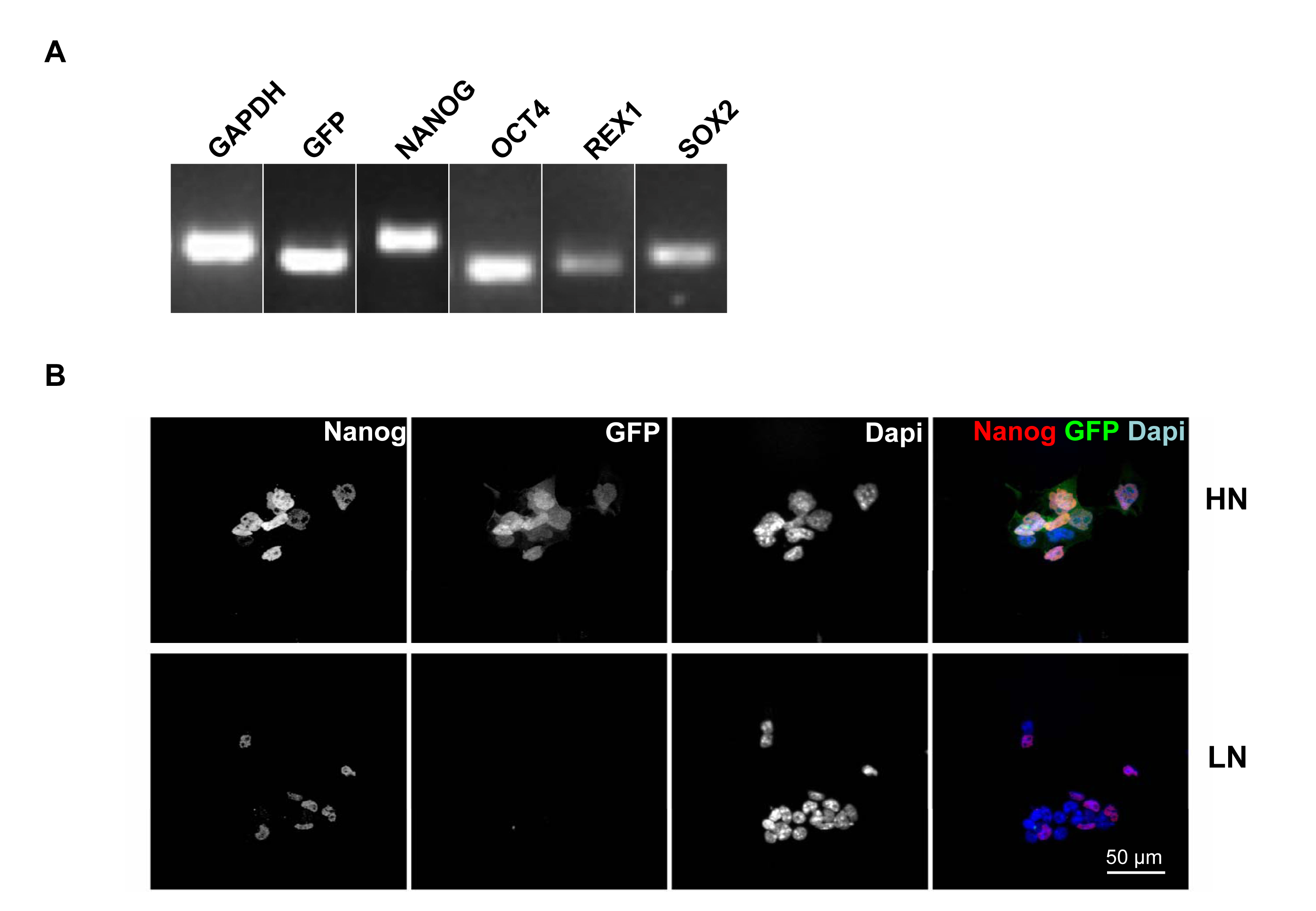

Supplement: Figure S1 — Pluripotency marker expression in P19 EC and TNGA ES cells. (A) RT-PCR data showing that cultured undifferentiated P19 cells express a set of pluripotency genes (Nanog, Oct4, Rex1, Sox2) that are also characteristic of the undifferentiated ES cells. RNA was extracted from P19OTOY cells and gene expression analysed using a two-step semiquantitative RT-PCR reaction (for details, see Materials and Methods). The expression levels of pluripotency markers are shown. Expression of GAPDH (cycle 28), GFP (cycle 32), Nanog (cycle 36), Oct4 (cycle 32), Rex1 (cycle 40), and Sox2 (cycle 32) were detected. (B) Correlation of Nanog expression and Nanog-GFP expression in TNGA ES cells. HN and LN cells were sorted and stained for Nanog expression (red). Although there is some expression of Nanog in the LN population, it is more heterogeneous and lower than in the HN population. The green channel shows the Nanog-GFP reporter, and the blue channel shows DAPI staining. (0.75 MB TIF) [file pbio.1000149.s001.tif]

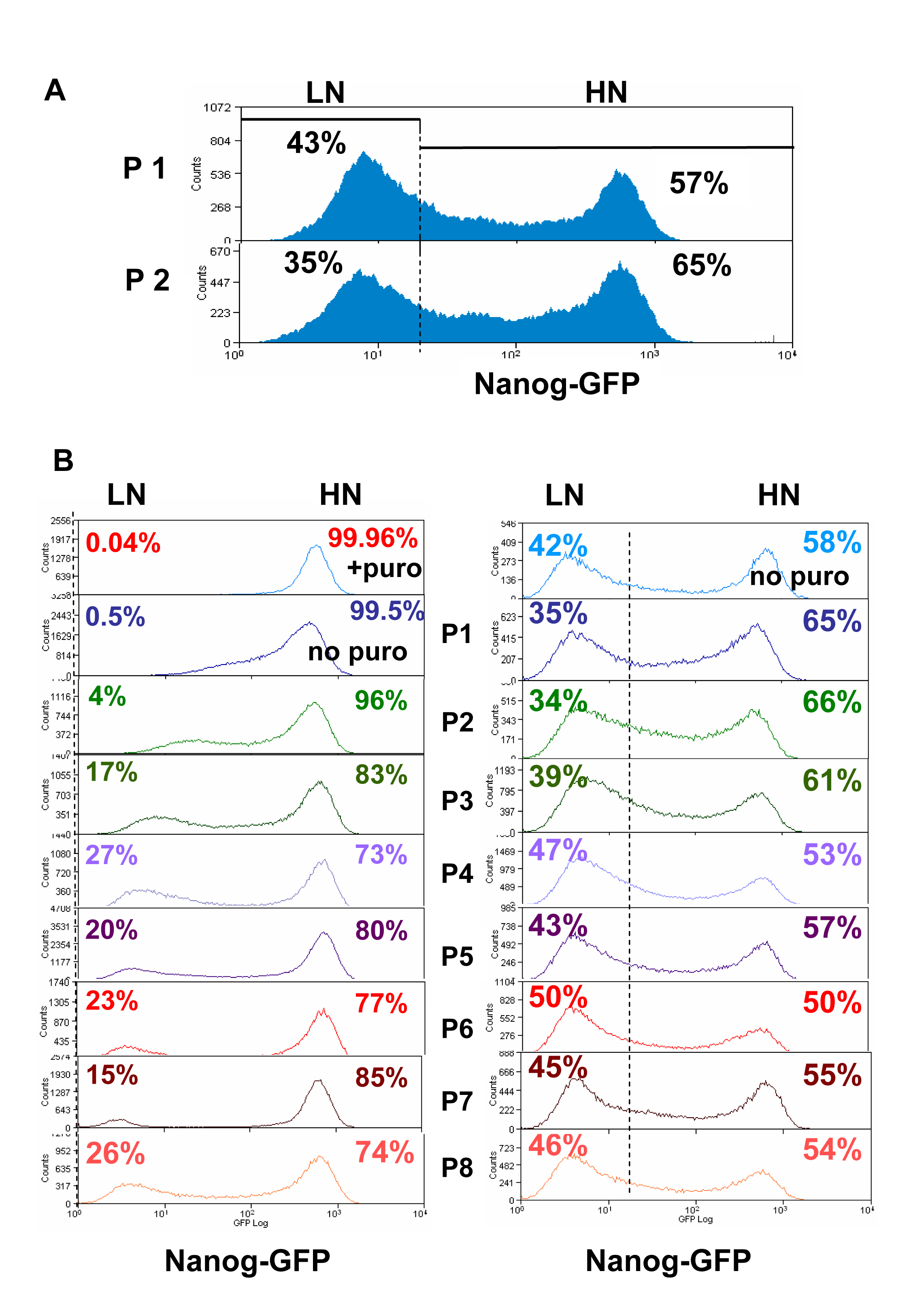

Supplement: Figure S2 — Recovery and stability of the TNGA ES cell population following puromycin selection. (A) Profile of two consecutive passages from a steady-state population of TNGA ES cells grown for several weeks without puromycin treatment. Notice that under these conditions, cells accumulate in the LN. Compare with Figure 1A. (B) Cells from the culture shown in (A) were treated with puromycin (left column) for three successive passages. The treatment selects for cells expressing high levels of Nanog-GFP and results in the elimination of cells that do not express Nanog-GFP. After three passages, puromycin selection was removed, and Nanog-GFP expression was monitored in the population over eight consecutive passages by flow cytometry. Notice that following the puromycin treatment, the majority of the cells in the culture are in the HN peak (99.96%) and that the relative ratio of LN cells progressively increases until the third/fourth passage, when the distribution stabilizes with a LN population between 15%–25% of the total. This distribution is comparable to the one noted in Chambers et al. [25] (and see Figure 1) and persists for four more passages (P5–P8) thereafter, suggesting that this is a steady-state distribution. For comparison, we show the culture from (A) grown in parallel, without selection in the same medium the cells were before the selection (right column). Long-term culture without selection leads to the increase of cells in the LN peak. (0.95 MB TIF) [file pbio.1000149.s002.tif]

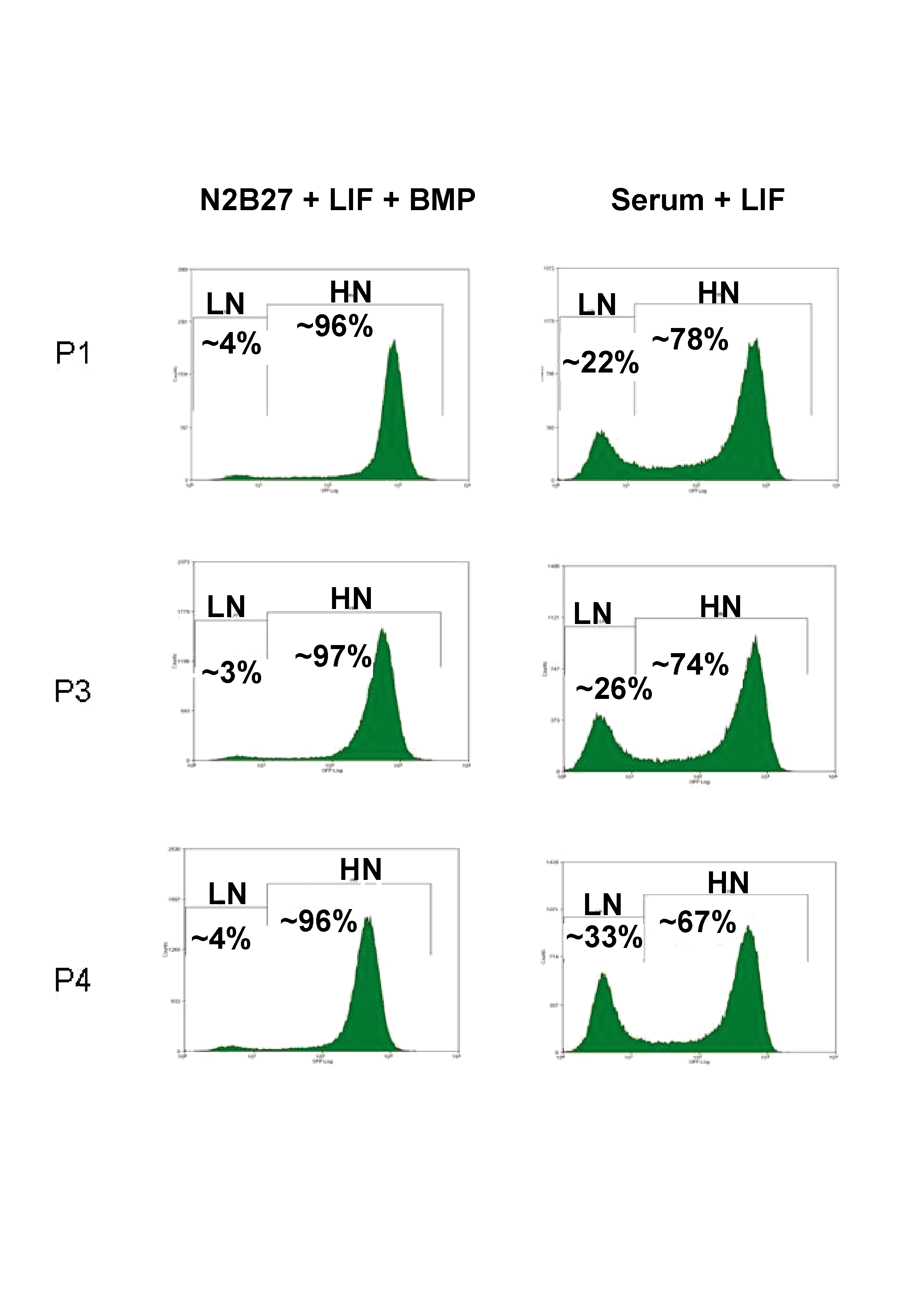

Supplement: Figure S3 — Culture conditions determine the ratio of LN/HN subpopulations in TNGA ES cells. FACS profiles (GFP expression) of TNGA cells grown in serum-free (LIF+BMP in N2B27) (left column) or in serum+LIF (right column) culture condition during four passages. In the serum-free condition, only 3%–4% of the whole population of TNGA cells is in the LN state, whereas in serum-containing medium, the proportion of cells with a low level of Nanog expression varies between 22% and 33%. Notice the stability of both profiles over time (steady state) and the similar position of the LN/HN peaks between the two conditions. (0.43 MB TIF) [file pbio.1000149.s003.tif]

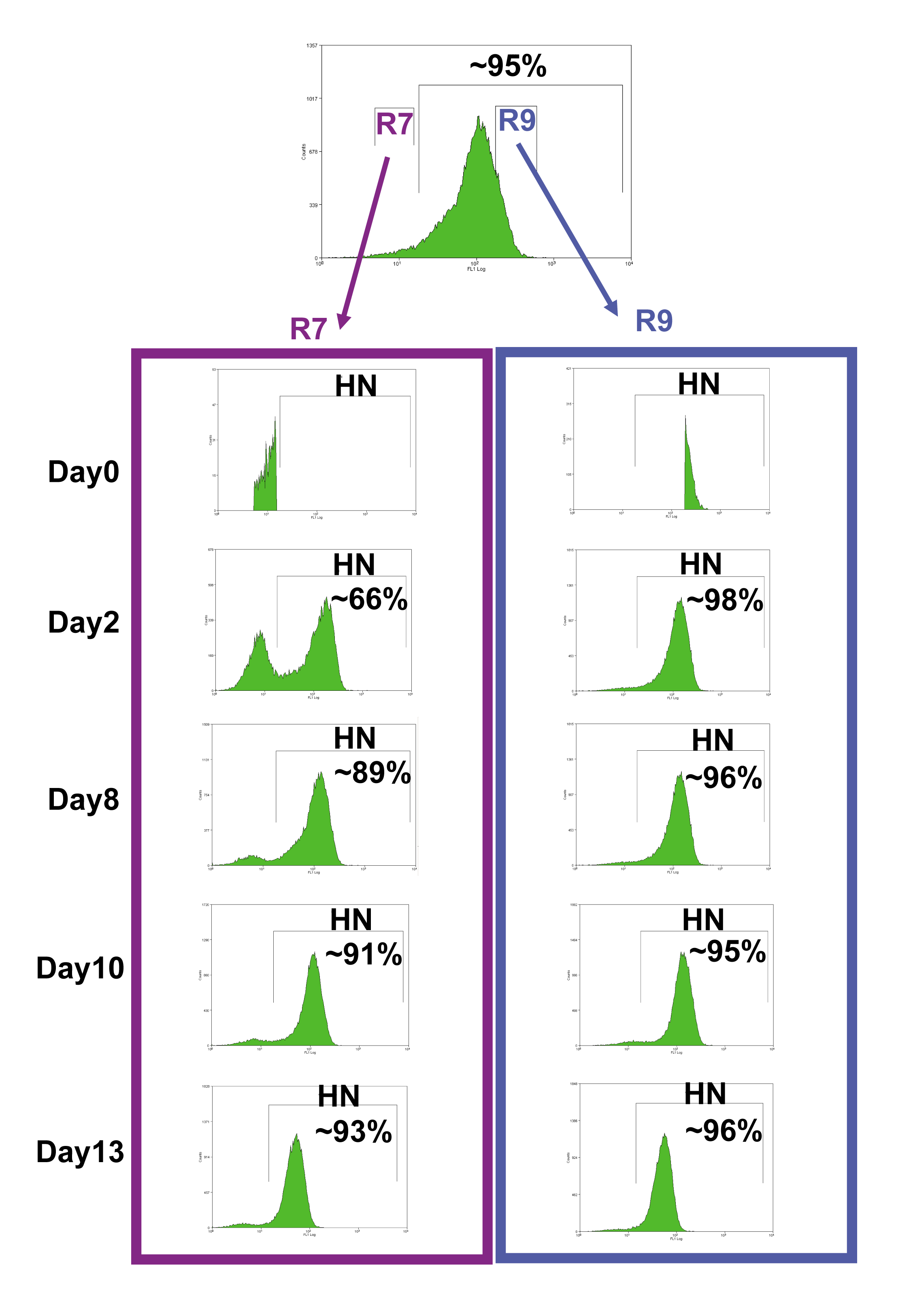

Supplement: Figure S4 — Reconstitution of the distribution of Nanog/YFP expression from the outliers in P19OTOY cells. LN (R7) (autofluorescence level of YFP expression) and HN (R9) (high level of YFP expression) subpopulations of P19OTOY cells were FACS sorted and subcultured in serum with LIF-containing medium. Periodically, FACS profiles of the samples were taken. Two days after culture, the LN population exhibited a clear bimodal distribution. Over time, it is possible to see how the population evolves towards the original distribution in which the LN peak is diffused by the tail of the HN peak, perhaps reflecting the existence of an occupied transition state between the HN and the LN peaks. By day 8 and certainly by day 10, one can see the population has equilibrated. We do observe some variability in the definition of the LN peak in the P19 cells, highlighting that the dynamic range is an important variable in the definition of the states. (0.34 MB TIF) [file pbio.1000149.s004.tif]
