# Supplementary material for: Regulated Fluctuations in Nanog Expression Mediate Cell Fate Decisions in Embryonic Stem Cells
Source: PLoS Biol. 2009 Jul 7;7(7):e1000149. doi: 10.1371/journal.pbio.1000149 (PMC2700273; doi:10.1371/journal.pbio.1000149)
Supplement: Protocol S1 — Details of the continuous (deterministic) and stochastic (discrete) models. (0.07 MB DOC) [file pbio.1000149.s005.doc]

**Protocol S1** **Details of the model; continuous and stochastic versions**

**Continuous model**

We consider a genetic circuit, shown in **Fig 3A** of the main text, involving one of the main factors that are known to maintain stemness, namely Nanog (*N*). The circuit includes a second factor that interacts with Nanog via a mutual activation motif, and that could be considered to be Oct4 (*A*). Besides their mutual activation, these two factors are known to positively regulate themselves [16,19,20]. Furthermore, there is evidence that sufficiently high levels of Oct4 *repress* Nanog [14,33]. We represent the latter process by including a third factor, *A^*^,* representing high levels of Oct4, which repress Nanog enzymatically. The dynamical behavior of the concentrations of these three factors can be described by the following three-dimensional differential-equation model:

$\begin{matrix} \frac{dN}{dt}=\alpha_{n}+\frac{\beta_{n}N^{n}}{k_{n}^{n}+N^{n}}-\lambda A^{*}N-\gamma_{n}N \\ \frac{dA^{*}}{dt}=\frac{\beta_{x}A^{p}}{k_{x}^{p}+A^{p}}-\gamma_{x}A^{*} \\ \frac{dA}{dt}=\alpha_{a}+\beta_{a}AN-\gamma_{a}A \end{matrix}$ (S1)

Here we have made the following assumptions:

- Activation of Nanog by Oct4 is saturated for all levels of Oct4. Therefore we ignore any influence of *A* on the expression of *N* in the first equation above.
- Oct4 activation by Nanog and by Oct4 itself is far from saturation. Therefore the regulated expression term in the *A* equation is linear on the product *AN*.
- The repression of Nanog by Oct4 only kicks in for large enough levels of the latter, as established by a relatively large value of *k_x_* (see parameter table below) in the expression term of the *A^*^* equation.

Furthermore, we assume that the third factor *A^*^* evolves on a time scale much smaller than those of Nanog and Oct4, which allows us to consider that the time derivative of *A^*^* is instantaneously zero. This leads to the simplified two-dimensional model that is shown in **Box 1** of the main text. A set of parameters that leads to the excitable dynamics reported in this paper is given in the table below.

| *α_a_* | 0.005 molec/s | *k_n_* | 3750 molec |  |  |
| --- | --- | --- | --- | --- | --- |
| *α_n_* | 0.1 molec/s | *k_x_* | 15000 molec | *λ* | 2·10^-7^ (molec·s)^-1^ |
| *β_a_* | 8·10^-9^ (molec·s)^-1^ | *γ_a_* | 3·10^-5^ 1/s | *n* | 2.0 |
| *β_n_* | 2.44 molec/s | *γ_n_* | 3.6·10^-4^ 1/s | *p* | 1.5 |
| *β_x_* | 50 molec/s | *γ_x_* | 0.01 1/s |  |  |

Parameters of the deterministic model

**Discrete (stochastic) model**

Noise can be added in a heuristic way to the differential-model introduced above, as described in **Box 1** of the main text, in order to represent the effects of random fluctuations in the excitable dynamics of the network. Alternative, we can represent intrinsic fluctuations in the biochemical reactions underlying the circuit by writing down these reactions explicitly, and modeling their dynamics directly by means of a Monte Carlo simulation algorithm such as Gillespie’s first reaction method [71].

A list of reactions that could underlie the circuit shown in **Fig3A** of the main text is:

Here, the rates of the bimolecular reactions are scaled with the cell volume [71], and those of the transcription reactions are multiplied by the same factor. This accounts for a global rescaling of the cell size, and consequently a global change of the noise level [65]. In the results presented in **Fig 3** of the main paper, the volume factor is considered to be 1, while in **Fig 7** of the main text, the three noise levels correspond to volume factors equal to 1, 0.5 and 0.2.

The last two reactions above correspond to regulated transcription of *N* and *A*, which are assumed to follow a Hill kinetics of the form:

$$f(N,k_{14},k_{n},n)=\frac{k_{14}\Omega N^{n}}{\left(k_{n}\Omega\right)^{n}+N^{n}}, g(N,k_{15},k_{x},p)=\frac{k_{15}\Omega N^{p}}{\left(k_{x}\Omega\right)^{p}+N^{p}}$$

Writing down the kinetic equations corresponding to the reactions listed above, and adiabatically eliminating the dynamics of the mRNAs, which are assumed to be much faster that those of the proteins, one can reach the differential equations (S1) above, with deterministic coefficients given by the reaction rates as follows:

$$\begin{matrix} \alpha_{n}=\frac{k_{1}k_{3}}{k_{8}}, \beta_{n}=\frac{k_{14}k_{3}}{k_{8}}, \gamma_{n}=k_{11} \\ \alpha_{a}=\frac{k_{21}k_{5}}{k_{10}}, \beta_{a}=\frac{k_{6}k_{5}}{k_{10}}, \gamma_{a}=k_{13} \\ \beta_{x}=\frac{k_{4}k_{15}}{k_{9}}, \lambda=k_{7}, \gamma_{x}=k_{12} \end{matrix}$$

Assuming reasonable values for the rates of translation *k_3_*, *k_4_*, and *k_5_* (equal to 0.2 transcripts/s) and for the mRNA degradation rates *k_8_*, *k_9_*, and *k_10_* (equal to 0.005 1/s), and taking into account the values of the deterministic parameters given in the previous table, one can determine the necessary values of the reaction rates, given in the table below.

| *k_1_* | 0.00375 s^-1^ | *k_7_* | 1.3·10^-7^ (molec·s)^-1^ | *k_13_* | 3·10^-5^ s^-1^ |
| --- | --- | --- | --- | --- | --- |
| *k_2_* | 1.875·10^-4^ s^-1^ | *k_11_* | 3.6·10^-4^ s^-1^ | *k_14_* | 0.091 s^-1^ |
| *k_6_* | 1.3·10^-10^ s^-1^ | *k_12_* | 0.01 s^-1^ | *k_15_* | 1.875 s^-1^ |

Parameters of the stochastic model
